# Are high-viscosity glass-ionomer cements inferior to silver amalgam as restorative materials for permanent posterior teeth? A Bayesian analysis

**DOI:** 10.1186/s12903-015-0108-5

**Published:** 2015-10-08

**Authors:** Steffen Mickenautsch

**Affiliations:** SYSTEM Initiative, Department of Community Dentistry, Faculty of Health Sciences, University of the Witwatersrand, 7 York Rd., Parktown/Johannesburg, 2193 South Africa

**Keywords:** Amalgam, High-viscosity glass-ionomer, Posterior teeth restoration, Bayesian probability

## Abstract

**Background:**

To develop a synthesis within a Bayesian probability framework of previously established evidence, in order to derive an overall conclusion about the hypothesis (H1): ‘High-viscosity glass-ionomer cements (HVGIC) are inferior to silver amalgam as (load bearing) restorative materials for permanent posterior teeth’.

**Methods:**

Following Bayesian method, the prior Odds that H1 is true (established from past uncontrolled clinical longitudinal and laboratory trials), the Likelihood Ratio incorporating new evidence (established from recent meta-epidemiological studies and systematic reviews of controlled clinical trials), as well as the posterior hypothesis Odds in view of the new evidence, were calculated.

**Results:**

The prior Odds that HVGICs are clinically inferior to amalgam as restorative materials in posterior permanent teeth in relation to the hypothesis that this is not so was 1.12 to 1. The Likelihood Ratio based on new evidence in favor the hypothesis was zero and the subsequent posterior Odds 0 to 1. Therefore, based on the new evidence, the Odds that HVGICs are clinically inferior to amalgam as restorative materials in posterior permanent teeth degreased from 1.12 to zero.

**Conclusion:**

The current evidence suggests lack of support for the hypothesis that high-viscosity glass-ionomer cements are inferior to silver amalgam as restorative materials for permanent posterior teeth. Should future research to this topic uphold the current findings, a wider range of clinical benefits for both patient and care provider, beyond appropriate restoration longevity for placing HVGIC based restorations may apply.

**Electronic supplementary material:**

The online version of this article (doi:10.1186/s12903-015-0108-5) contains supplementary material, which is available to authorized users.

## Background

The term ‘high-viscosity’ or ‘high-viscous glass-ionomer cement’ (HVGIC) has emerged within the scientific dental literature and is related in clinical studies specifically to the products Fuji IX (GC Corporation, Japan) or Ketac Molar (3 M ESPE, Germany) [1]. A definition of HVGICs in line with chemical characteristics such as the powder – liquid ratio or its compressive strength in comparison to other chemically cured glass-ionomers appears difficult due to contradictive in-vitro evidence [1]. However, HVGICs appear distinct from other (low) viscosity glass-ionomers (including Cermets) in their comparative clinical survival rate to that of conventional amalgam restorations. Meta-analysis results indicate a survival rate for HVGIC (Fuji IX; Ketac Molar) tooth restorations similar to that of amalgam but show significantly lower survival rates for “low-viscosity” GICs (Chelon Silver (= Cermet); Chem Fil; Fuji II) than for amalgam [2].

Glass ionomers, such as HVGICs, are reported to adhere primarily via calcium bonds to the mineral content of teeth [3] and thus provide an adaptive seal. As HVGIC’s leach fluoride ions into the adjacent tooth tissue, these materials are assumed to be capable of slowing the progression of carious lesions [4]. For these reasons, HVGICs are expected to be ideally suited for the management of dental caries. Additionally, they may simplify the tooth restorative procedure and enable the dentine-pulp complex to react against the caries process [5].

During a systematic review of clinical controlled trials, the survival rate of HVGIC restorations, placed using the atraumatic restorative treatment approach, in permanent posterior teeth in comparison to conventionally placed silver amalgam has been established [6]. This systematic review was further updated [7] and the detailed results, including additional results from Chinese trials, published by the authors [8, 9]. All published reports of this systematic review indicated no differences between HVGIC and amalgam, beyond the play of chance (p > 0.05) in the permanent dentition after four and six years for single and multiple surface tooth restorations, respectively, and no differences after three years for single and multiple surface restorations in primary teeth [6, 7, 10]. The results from Chinese trials were confirmatory of these findings [8].

The systematic review findings are in disagreement with results from one comprehensive, non-systematic literature review by Manhart et al. [11]. The conclusion of this review was that glass-ionomers were generally inferior to amalgam for placing restorations in posterior teeth. This review extracted the annual failure rates of different restoration types, including amalgam and glass-ionomers, from mainly clinical cross-sectional and uncontrolled clinical longitudinal studies and calculated their mean with standard deviation and median values for naïve-indirect comparison by use of analysis of variances (ANOVA) [11].

Against this background, an empirical meta-epidemiological study was conducted in order to investigate whether trends and performance differences between conventional amalgam and direct HVGIC restorations in posterior teeth can be correctly inferred through naïve-indirect comparison of failure rates from uncontrolled longitudinal clinical studies [1]. Based on the study’s result, the null-hypothesis that trends and performance differences inferred from naïve-indirect-indirect comparison based on evidence from clinical uncontrolled longitudinal studies and from direct comparisons based on randomised control trial (RCT) evidence, concerning conventional amalgam versus direct HVGIC restorations, have similar direction and magnitude, was rejected. It was further concluded that naïve-indirect comparison of failure rates from uncontrolled longitudinal clinical studies are unsuitable for clinical inference, particularly in regard to the clinical HVGIC efficacy for placing direct tooth restorations.

In addition to uncontrolled longitudinal clinical studies, laboratory trial results are sometimes used as basis for clinical inference and recommendations for daily dental practice. Based on laboratory evidence, glass-ionomers are traditionally considered as unsuitable for clinical use as a permanent filling material in the posterior dentition due to in-vitro measured poor mechanical properties [12, 13]. Specifically, in-vitro measured low material strength and wear resistance have been stated as reasons why glass-ionomers cannot rival amalgam as truly universal posterior restorative material [14].

A meta-epidemiological study was conducted in order to test the null-hypotheses whether the results from laboratory trials concerning HVGICs versus amalgam indicate similar effect direction and magnitude as results from clinical controlled trials concerning HVGICs versus amalgam restorations placed in permanent posterior teeth [10]. The results of this study showed that the effect direction and magnitude are not similar and that, similar to the investigation concerning the naïve-indirect-indirect comparison method based on evidence from clinical uncontrolled longitudinal studies [1], the null-hypotheses had to be rejected. In addition, this study raised reasons for doubt regarding the general suitability of laboratory trials for clinical inference [10].

After the conduct and reporting of systematic review [8, 9] and meta-epidemiological study [1, 10] results regarding the clinical efficacy of HVGICs versus amalgam as the current restorative gold standard, an integrative analysis of all evidence combined to the topic has still been missing. For this reason, the aim of this study was to present a synthesis of previously established evidence within a Bayesian framework, in order to derive an overall conclusion about the Odds in regard to the hypothesis that HVGIC’s are inferior to silver amalgam as restorative materials for permanent posterior teeth.

## Method

### Bayesian framework

Two hypotheses were generated:

(i) H1: HVGICs are clinically inferior to amalgam as restorative materials in posterior permanent teeth;
(ii) H2: HVGICs are clinically not inferior to amalgam as restorative materials in posterior permanent teeth.

Following Bayesian method, the prior Odds of hypothesis H1 (relative to H2) based on previous evidence, the Likelihood Ratio incorporating new evidence, as well as the posterior Odds of hypothesis H1 (relative to H2) in view of the new evidence, were calculated.

The prior Odds (Odds_Pre_) was calculated from the ratio of the probability that H1 is correct, P(H1), to the probability that H2 is correct, P(H2), i.e.: Odds_Pre_ = P(H1)/P(H2); with P(H2) = 1 – P(H1). All probabilities were calculated as the ratio of the number of hypothesis supporting events (n) to the total number of evaluated events (N) based on previous evidence from naïve-indirect-indirect comparison of results from clinical uncontrolled longitudinal trials and from laboratory trials.

The Likelihood Ratio (LR) was calculated from the ratio of the probability of H1 according to new evidence, P(E|H1) to the probability of H2 according to new evidence, P(E|H2), i.e.: LR = P_n_(E|H1)/P_n_(E|H2) × P_n+1_(E|H1)/P_n+1_(E|H2). The new evidence (E) was established from empirical study results (labelled as ‘Evidence 1’) [1, 10], and systematic review evidence (labelled as ‘Evidence 2’) [8, 9].

The posterior Odds (Odds_Post_) were calculated by multiplication of the prior Odds with the Likelihood Ratio.

An assessment of sufficient statistical power due to sample size was conducted. If sample sizes were too small the event results would lack sufficient statistical power in order to detect meaningful differences (beyond the play of chance) between effect estimates and thus would erroneously favor hypothesis H2. For this reason, all extracted events were analyzed for sufficient statistical power.

The assessment was based on the following assumptions:

(i) Risk of type I error (risk of falsely detecting a difference), α = 5 %;
(ii) Risk of type II error (risk of not detecting a true difference), β = 20 %;
(iii) Power to detect a 10-percentage point difference in the failure rate between HVGIC and amalgam restorations.

The assumption of 10-percentage point difference in the failure rate was chosen in line with the work by Taifour et al. [15] concerning placed HVGIC restorations in permanent teeth versus amalgam. The analysis was conducted by calculating the needed sample size per group (N_P_) with an assumed test group (HVGIC) event rate (P_1_ in %) that is 10 percentage points higher than that of the control group (P_2_ in % /Amalgam) using the formula by Pocock [16]:

1. N_P_ = {[P_1_(100 − P_1_) + P_2_(100 − P_2_)]/(P_2_ – P_1_)^2^} x *ƒ*(α, β), with:
  - P_1_ = P_2_ + 10 %
  - Control group event rate = n/N
  - ƒ(α, β) = ƒ(0.05, 0.20) = 7.9 [16]

The event rate of the control group (P_2_) was calculated from the number of failed amalgam restorations (n) in relation to the total number of restorations evaluated (N) that were extracted from the clinical trials.

Datasets with a combined sample size (test- and control group / N_T_) that was lower than twice the needed sample size per group (2xN_P_) were excluded from analysis. For datasets from Evidence 2 [8, 9], where more than one dataset per trial was reported, the next dataset with sufficient sample size and longest follow-up period was chosen. All datasets for sensitivity analyses are presented in Additional file 1: Table S1.

### Prior odds of hypothesis H1 relative to H2

A total of 17 events were observed from naïve-indirect comparisons of clinical uncontrolled longitudinal trial results^1^ and from laboratory trial results [10]. The event outcome was reported as Odds ratio (OR) or Standardised Mean Difference (SMD) with 95 % Confidence interval (CI) (Additional file 1: Table S1).

Events observed from naïve-indirect comparisons of clinical uncontrolled longitudinal trial results originated from two groups of trials: one group investigating HVIGICs and the other amalgam. All trials were identified through a systematic literature search in the PubMed/Medline database (Date of search: September 25, 2012) following a simple, systematic search strategy, including the search terms: “atraumatic restorative treatment” for longitudinal studies investigating HVGIC and the string of MeSH search terms "Dental Amalgam"[Mesh] AND "Dental Restoration, Permanent"[Mesh] for longitudinal studies investigating amalgam. The search period was limited to publications from 2002/01/01 to 2012/09/25. Trial inclusion criteria were: (i) Prospective clinical one-arm study (uncontrolled longitudinal study investigating either direct HVGIC or conventional amalgam restorations) or quasi-one-arm study (two-arm study that did not compare HVGIC with amalgam restorations, but included either HVGIC or amalgam as one of the study arms); (ii) Minimum 12-month follow-up period; (iii) Investigated cavity type Class I or II in permanent posterior teeth (Tunnel restorations not included); (iv) Publication language: English; (v) Study outcome: restoration failure [1].

Events observed from non-clinical, laboratory investigations originated from trials that established the material characteristics of HVGICs with silver amalgam as control. The trials were identified on basis of a systematic literature search (Dates of search: September 12 and 14, 2014), comprising a search of the databases: CENTRAL accessed via Cochrane Library; MEDLINE accessed via PubMed; Biomed Central; Database of Open Access Journals (DOAJ); IndMed; OpenSIGLE and Google Scholar. Trial inclusion criteria were: (i) Articles published in English; (ii) Full reports of prospective clinical controlled (including randomised control trials and non- randomised control trials) and laboratory trials (including: in-vitro; in-vivo on animal tissues); (iii) Head-to-head comparison of high-viscosity glass-ionomers (HVGIC) versus amalgam; (iv) Longest follow-up period reported per trial; (v) Relevance to tooth restorations in posterior teeth of the permanent dentition; (vi) Computable data reported [10]. Further detailed information regarding both systematic literature searches have been presented elsewhere [1, 10].

Events with 95 % Confidence intervals that indicated a statistically significant higher effect size in favour of amalgam were considered as evidence in support of hypothesis H1 (n_H1_) and events whose 95 % Confidence intervals indicated no statistically significant difference between HVGICs and amalgam, as well as events that indicated a statistically significant higher effect size in favour of HVGIC were considered as evidence in support of hypothesis H2 (n_H2_). All events are presented in Additional file 1: Table S1. The prior Odds of hypothesis H1 relative to H2 were calculated using the following mathematical steps:

(2) P(H1) = n_H1_/N and P(H2) = n_H2_/N [1, 10]
(3) Odds_Pre_ = P(H1)/P(H2)

### Likelihood Ratio of strength of evidence in favour of hypothesis H1 relative to hypothesis H2

All studies concerning naïve-indirect-indirect comparison based on evidence from clinical uncontrolled longitudinal trials [1] and laboratory trial results [10], established only poor and unreliable bases for clinical inference. Therefore, the probabilities, derived from Evidence 1 were assumed to be equivocal for hypothesis H1 and H2, i.e.: P(H) = 0.50.

A total of three events related to restorations placed in posterior permanent teeth (Additional file 1: Table S1) were observed from RCTs. The trials were identified on basis of a systematic literature search up to January 2012 [8, 9] including the databases MEDLINE accessed via PubMed; CENTRAL accessed via Cochrane Library; Open access sources: Biomed Central, Database of Open Access Journals (DOAJ), OpenJ-Gate; Regional databases: Bibliografia Brasileira de Odontologia (BBO), Literatura Latino-Americana e do Caribe em Ciências da Saúde (LILACS), IndMed, Sabinet, Scielo; Grey-Literature sources: Scirus (Medicine), OpenSIGLE, Google Scholar [9]; as well as Chinese Biomedical Literature Database (CBM), China National Knowledge Infrastructure (CNKI, formerly China Academic Journals), VIP Information and WanFang Data [8]. Further detailed information regarding the systematic literature search have been presented elsewhere [8, 9]. Because the systematic review reported data at all follow-up intervals per trial, only events with the longest follow-up period per trial were selected for analysis, in order to avoid data duplication. Where available, results from meta-analyses were given selection priority over that of single datasets [8, 9]. The result of each event was reported as Odds ratio (OR with 95 % CI). Events whose 95 % Confidence intervals indicated a statistically significant higher effect size in support of amalgam were considered as evidence in favour of hypothesis H1 (n_H1_) and events whose 95 % Confidence intervals indicated no statistically significant difference between HVGICs and amalgam or indicated a statistically significant higher effect size in favour of HVGIC were considered as evidence in support of hypothesis H2 (n_H2_). The Likelihood Ratio of strength of evidence in support of hypothesis H1 relative to hypothesis H2 was calculated using the following steps:

(4) P_1_(E_1_|H1) =  assumed to be 0.50
(5) P_2_(E_2_|H1) = n_H1_/N_2_and P_2_(E_2_|H2) = n_H2_/N_2_
(6) LR = P_1_(E_1_|H1)/P_1_(E_1_|H2) × P_2_(E_2_|H1)/P_2_(E_2_|H2)

## Results

**Fig. 1:**
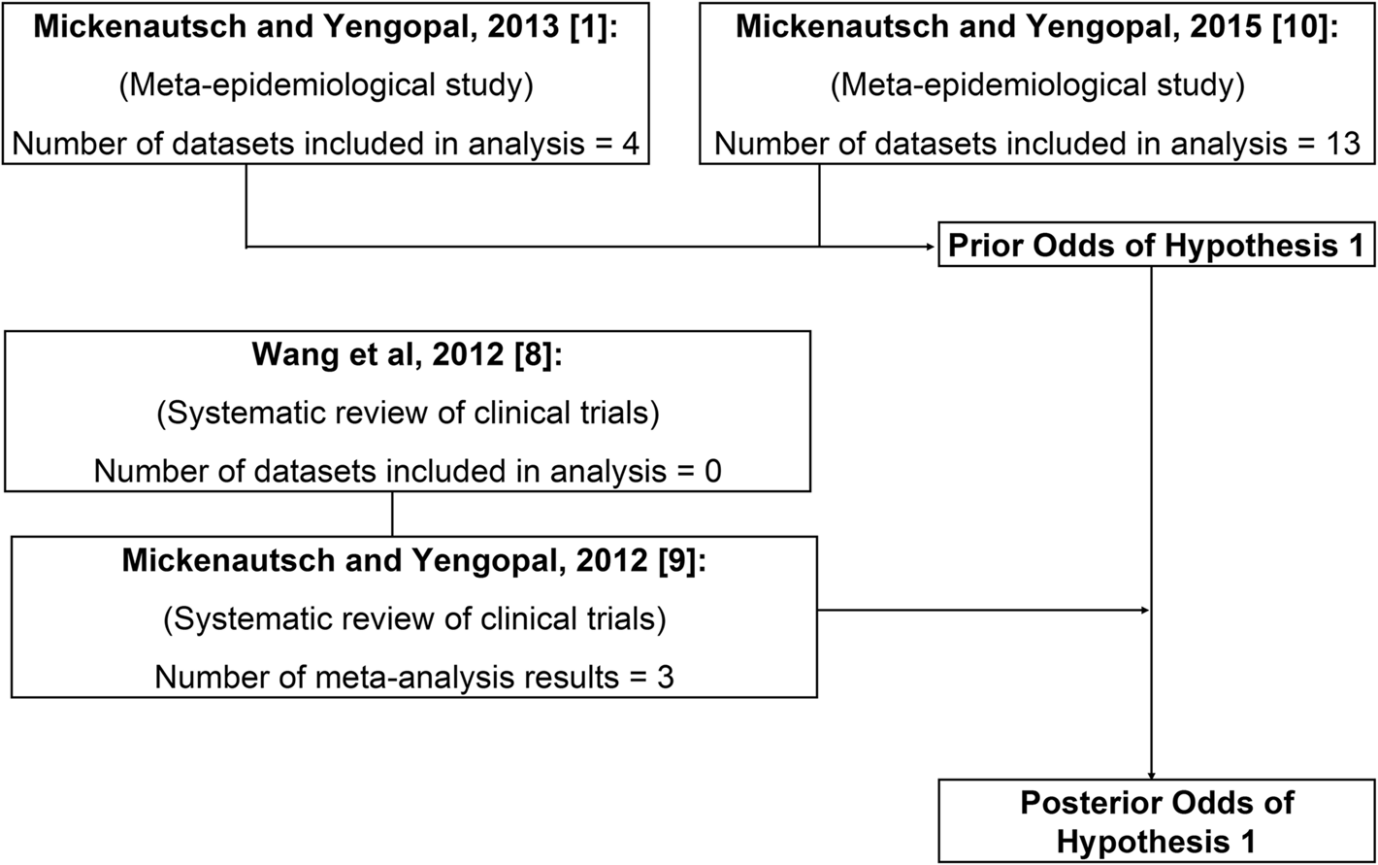
Flow chart of evidence included in the Bayesian analysis. Number of meta-analysis results and datasets included in analysis, after assessment for sufficient statistical power

Figure 1 shows the sources of evidence that were included in the Bayesian analysis. Nine and eight out of 17 events from uncontrolled clinical longitudinal and laboratory studies (n/N) were found in support of the hypothesis H1 and H2, respectively [1, 10]. Therefore, the prior Odds (Odds_Pre_) that HVGICs are clinically inferior to amalgam as restorative materials in posterior permanent teeth (H1) in relation to the hypothesis that this is not so (H2) was 1.12 (Table 1).

**Table 1:** Bayesian probability analysis

1. Hypotheses		
H1: HVGICs are clinically inferior to amalgam as restorative materials in posterior permanent teeth		H2: HVGICs are clinically not inferior to amalgam as restorative materials in posterior permanent teeth
2. Prior Odds of hypothesis H1 relative to H2		
a. Evidence	Evidence 1 [1, 10]	
	P(H1) = n_H1_ / N = 9 out of 17 = 0.53	
	P(H2) = n_H2_ / N = 8 out of 17 = 0.47	
	Odds_Pre_ = P(H1) / P(H2)	
	Odds_Pre_ = 0.53 / 0.47	
b. Odds_Pre_	=1.12	
3. Likelihood Ratio (LR) in support of hypothesis H1 relative to hypothesis H2		
a. New Evidence	Evidence 1 [1, 10]	
	P_1_(E_1_|H1) = 0.50	
	P_1_(E_1_|H2) = 0.50	
	Evidence 2 [8, 9]	
	P_2_(E_2_|H1) = n_H1_ / N_3_ = 0 out of 3 = 0	
	P_2_(E_2_|H2) = n_H2_ / N_3_ = 3 out of 3 = 1.00	
	LR = P_1_(E_1_|H1)/ P_1_(E_1_|H2) × P_2_(E_2_|H1)/ P_2_(E_2_|H2)	
	LR = (0.50/0.50) × (0/1.00)	
b. LR	=0	
4. Posterior Odds of hypothesis H1 relative to H2		
a. Calculation	Odds_Post_ = Odds_Pre_ × LR	
	Odds_Post_ = 1.12 × 0	
b. Odds_Post_	=0	

New evidence was obtained from investigations concerning the suitability of naïve-indirect comparison of results from uncontrolled clinical longitudinal studies [1] and laboratory trials [10] for clinical inference. The results of both were found to be inconsistent with that of clinical randomised control trials (RCT) and thus were judged to provide only uncertain evidence for either hypothesis. For that reason, the probabilities, P(H), for hypothesis H1 and H2 based on Evidence 1 were estimated to be 0.50. In addition, the evidence from a systematic review of clinical controlled trials was included (Evidence 2) [8, 9]. In the systematic review report, a total of zero and three out three events from clinical control trials (n/N) were found in support of the hypothesis H1 and H2, respectively (Evidence 2). Data of the three events was obtained from two meta-analyses and eight single datasets (Additional file 1: Table S1). Consequently, the calculated Likelihood Ratio (LR) was zero (Table 1).

**Fig. 2:**
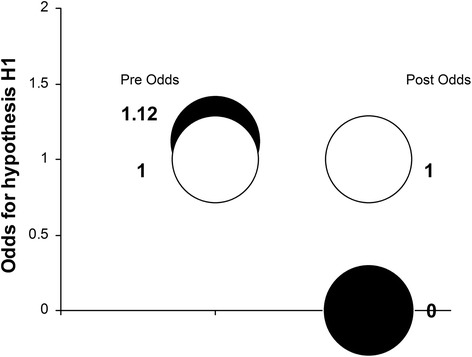
Odds in support of hypothesis H1. The black circles’ distance from the white circles on the vertical scale represent graphically the Odds for H1 in relation to H2 (=1.00)

Multiplication of the Likelihood Ratio (LR) with the prior Odds (Odds_Pre_) lead to the posterior Odds (Odds_Post_) that hypothesis H1 is true (relative to H2) to be zero. Therefore, based on the new evidence [1, 8–10], the Odds that HVGICs are clinically inferior to amalgam as restorative materials in posterior permanent teeth degreased from 1.12 to zero (Table 1). The results were graphically presented in Fig. 2.

Based on the applied criteria for sufficient statistical power [15, 16] the results of 22 single datasets from Evidence 1 and one meta-analysis and eight single datasets from Evidence 2 were excluded. The results from laboratory studies were all included, as these did not originate from clinical investigations (Additional file 1: Table S1).

## Discussion

The aim of this study was to present a synthesis of previously established evidence, in order to derive an overall conclusion about the Odds in regard to the hypothesis that HVGIC’s are inferior to silver amalgam as restorative materials for permanent posterior teeth. For this purpose, a Bayesian framework was used.

### Limitations of method

The main objection to Bayesian analysis is that prior probabilities of hypotheses, i.e. before consideration of any evidence, were traditionally established from pure subjective assumptions [17]. Such assumption would have directly affected the prior Odds (Odds_Pre_), which is used for calculation of the final analysis result (Odds_Post_ = Odds_Pre_ × LR). Within the context of this study, no subjective assumptions were utilized, thus the objection to the use of Bayesian analysis in this study does not apply. Instead of subjective assumptions, prior probabilities were established from evidence that was identified through systematic literature search [1, 10]. The results’ limitation need to be considered in line with the limitations of the literature searches, which were discussed in details elsewhere [1, 10]. These limitations were: both searches were restricted to English literature [1, 10], and one search used PubMed as the only database and only included literature listed during the period from 2002 – 2012 [1].

In addition, the results are further limited by the internal validity of all clinical trials that were included in the meta-epidemiological studies [1, 10] and systematic reviews [8, 9]. Trial validity assessment was included in both and judged as generally weak [1, 8–10].

The language restriction was justified on basis that treatment effect estimates from non-English studies are shown to be 16 % more beneficial (Ratio of estimates 0.84; 95 % CI: 0.74 – 0.97; *p* = 0.011) than that of results published in English [18] and thus may introduce some level of overestimation. Thus excluding non-English trials from the systematic literature searches might in some cases render its results more conservative. In contrast, language restricted meta-analyses, compared to language inclusive meta-analyses did not differ in their effect size estimates (ROR 50.98; 95 % CI: 0.81–1.17) [19]. For these reasons, the failure to include non-English trials may not have biased the current results. While the search in one main database (PubMed) may have limited the results in one study [1] its 10-year publication limit may have reduced the risk of chronological bias as no RCTs that provided direct comparison between HVGIC and amalgam restorations (used as control group in that study) before that period could be identified.

Bayesian analysis by multiplication of probability ratios, P_n_(H1)/P_n_(H2) x P_n+1_(H1)/P_n+1_(H2), from different evidence sources is based on the assumption that these sources are independent from each other. Evidence 1 [1, 10] may not be regarded as strictly independent from Evidence 2 [8, 9], as the result of the latter served as control data for the former [1, 10]. However, the results of both empirical studies let to a 50/50 assumption in support for either hypothesis and rendered the product, P_1_(E_1_|H1) / P_1_(E_1_|H1) x P_2_(E_2_|H1) / P_2_(E_2_|H1) as 1.00, thus did not affect mathematically the calculation of the Likelihood Ratio (Table 1).

Neither the results from possible naïve-indirect comparisons of uncontrolled laboratory trials nor the results from case reports or conclusions from non-systematic literature reviews (investigating either HVGICs or amalgam) were included in the Bayesian analysis. This may have reduced the Odds_Pre_ - value but in turn will also have increased the validity of the prior Odds, as it may have reduced the level of bias risk and subjectivity that these types of study methods carry.

### Analysis results

The Bayesian analysis results suggest that the posterior Odds for the hypothesis that HVGICs are inferior to silver amalgam as restorative materials for permanent posterior teeth are zero (H1). Such results remain subject to revision on basis of future evidence that may or may not corroborate the current evidence. However, the current results from new evidence (E), identified through several systematic literature searches [1, 8–10] and under consideration of any prior evidence to this topic reduced the Odds to zero. The result can be explained on the basis that all data from Evidence 1 had to be considered invalid resulting in an equivocal 50/50 probability that either hypothesis is true. Results from Evidence 2 indicated no event in support of hypothesis H1. Therefore, according to Bayesian calculus, the P_2_(E_2_|H1)/ P_2_(E_2_|H2) quotient is derived by division of zero, thus rendering all subsequent multiplications with that quotient zero, as well.

If HVGIC restorations placed in permanent posterior teeth exhibit indeed no inferior clinical efficacy than silver amalgam (as still current gold standard for posterior tooth restorations) then their use as valid restorative treatment option may be justified. Such treatment option may have additional clinical benefit, besides its apparent lack of any higher restoration failure rates [6–9]: It has been reported that HVGICs are most suitable for tooth restoration after minimally-invasive cavity preparation, i.e. hand excavation of infected carious tooth tissue during the atraumatic restorative treatment approach [20]. Such restorative treatment that combines the use of HVGIC with cavity preparation by hand excavation has been clinically shown to generate smaller tooth restorations at the same clinical indications that would result in larger dental fillings, if amalgam had been placed by use of conventional cavity preparation with high-speed drilling instead [21]. The smaller HVGIC restorations have further been associated with less pain during placement in comparison to conventional amalgam restorations [22] and thus higher patient comfort during treatment with subsequent reduced levels of dental patient anxiety in adults [23]. A reduced level of patient anxiety may be associated with low operator stress levels, as high patient anxiety has been shown as one of the main stressors in daily dental practice [24]. In addition, HVGIC restorations, placed using the atraumatic restorative treatment approach, have been found to be more cost-effective than conventionally placed amalgam [25, 26] or composite restorations [26].

Against the background of such potential benefits and the Minamata Convention on Mercury’s call for a phase-down approach to dental amalgam [27], HVGICs may be considered as possible amalgam alternative. However, the large number of excluded dataset and meta-analysis results is an indicator for remaining research gaps concerning HVGICs. These gaps were reported previously [28] and include a general lack of trials to many GIC related topics, as well as weak statistical power of existing trials due to small sample size. Such lack of statistical power was found to be further associated with wide confidence intervals of the effect estimates, which rendered trial results inconclusive. A particular need for more trials of suitable sample size was identified for establishing restoration longevity of HVGIC fillings placed, using hand-excavation following the atraumatic restorative treatment approach, in posterior permanent restorations in comparison to amalgam [28].

## Conclusions

While the current evidence suggests lack of support for the hypothesis that high-viscosity glass-ionomer cements are inferior to silver amalgam as restorative materials for permanent posterior teeth, further studies are needed. Should further research uphold the current findings, a wider range of clinical benefits for both patient and care provider, beyond appropriate restoration longevity for placing HVGIC based restorations may apply.

## Additional file

Additional file 1: Table S1. Extracted event data. (XLS 127 kb)
